# Latent Autoimmune Diabetes in Adults and a Continuous Glucose Monitoring Device: An Unfortunate Outcome

**DOI:** 10.7759/cureus.49141

**Published:** 2023-11-20

**Authors:** Rohit Gupta, Srujan Edupuganti, Irma Zamir, Adiraj Singh, Hemant T Thawani

**Affiliations:** 1 Internal Medicine and Pediatrics, Hurley Medical Center, Michigan State University, Flint, USA; 2 Endocrinology, Diabetes and Metabolism, Hurley Medical Center, Michigan State University, Flint, USA

**Keywords:** latent autoimmune diabetes in adults, obsessive-compulsive disorder, suicide, ocd, cgm, continuous glucose monitoring, lada

## Abstract

Latent autoimmune diabetes in adults (LADA) is a slow-progressing form of autoimmune diabetes. A 44-year-old man with a four-year history of diabetes mellitus (DM), obsessive-compulsive disorder (OCD), and panic disorder was admitted to the hospital for diabetic ketoacidosis. LADA was confirmed with positive GAD-65 antibody. His occupation involved random working days with several weeks off in between projects. During workdays, his insulin dosage required frequent adjustments due to lower blood glucose (BG) readings. Owing to the variable work schedule and constantly changing insulin needs, he was recommended a continuous glucose monitoring (CGM) device. Few days after starting on the CGM device, he was seen in the emergency department because of elevated BG. His home BG readings ranged from 80 to 408 mg/dL. He was getting frustrated with the fluctuating BG readings. At home, he remained agitated and endlessly checked his CGM device. After discharge, he would repeatedly call the endocrinology office with his BG readings with the insulin dose being adjusted accordingly. Few weeks later, the office received a call from his wife informing us that the patient had shot himself in the head. According to his wife, lately he had trouble sleeping, was very anxious, and often had panic attacks. He seemed to struggle with ever-fluctuating BG readings and was obsessed with incessantly changing numbers on his CGM device. Patients with Type 1 DM are at increased risk of mental health disorders and suicide forms a sizeable proportion of deaths in these patients. This case highlights the importance of mental health, especially underlying OCD as a prognostic factor in the management of diabetes with CGM devices.

## Introduction

Diabetes mellitus (DM) is a chronic, metabolic disease of inappropriately elevated blood glucose (BG) levels. Etiologically, DM has several subtypes which majorly include type 1 (T1DM), type 2 (T2DM), and gestational DM [1]. The two main subtypes T1DM and T2DM primarily result from lack of secretion (T1DM) and/or peripheral resistance (T2DM) of insulin. T1DM mostly presents early in childhood or adolescence, while T2DM commonly affects middle-aged to older adults with poor diet and lifestyle choices [2]. Latent autoimmune diabetes in adults (LADA) is an autoimmune DM with onset in adulthood. It has genetic, immunologic, and metabolic features of both T1DM and T2DM [3]. Hence, LADA is sometimes also referred to as type 1.5 DM [4]. These individuals, at diagnosis, clinically resemble T2DM by not requiring insulin treatment early in the disease course, although they have immunogenetic markers associated with T1DM. It is usually a slow-evolving form of autoimmune DM and accounts for 2-12% of all patients with adult-onset DM [5]. The American Diabetes Association (ADA) lists LADA as T1DM which is due to autoimmune pancreatic β-cell destruction usually leading to absolute insulin deficiency [6]. The Immunology for Diabetes Society (IDS) proposed these three criteria for the diagnosis of LADA [7]: 1) age usually ≥30 years, 2) positive titer for at least one of the four autoantibodies, and 3) has not been treated with insulin within the first six months after diagnosis (Table 1). Nonetheless, still there is no consensus on the diagnostic criteria for LADA due to slower progression of immune-mediated destruction of islet β-cells compared to T1DM even in the presence of autoantibodies, choice of treatments, and lack of severe hyperglycemia compared to T1DM [3]. Patients with T1DM use fingersticks for blood glucose monitoring (BGM) and a glucometer, and/or continuous glucose monitoring (CGM) device. CGM continually monitors BG levels providing real-time updates through a device attached to the patient's body with accurate BG levels. Patients with T1DM require strict control of BG levels which helps in optimizing their insulin regimen and avoiding episodes of hypoglycemia which can be readily achieved by CGM. Studies have demonstrated that the use of CGM provides the greatest glycemic benefit and improves safety in patients with nocturnal hypoglycemia and hypoglycemic unawareness [8]. Keeping in mind CGM is just a tool that like any other disease condition works the best in motivated patients in conjunction with compliance of treatment recommendations. CGM is also being regarded as the future of diabetic management. Mental health has always been a concern with any chronic medical condition including DM. Diabetes distress is a well-recognized entity since it was first described in the mid-1990s. It is estimated that patients with DM are 2 times more likely to have depression than the general population [9]. Some studies show a higher prevalence of diabetes in obsessive-compulsive disorder (OCD) individuals with early animal and genetic studies suggesting a possible role of insulin-signaling in the pathophysiology of OCD [10]. In this case report, we discuss an unfortunate outcome in our patient with LADA and underlying OCD after being started on a CGM device. 

## Case presentation

A 44-year-old man with a four-year history of DM was admitted to the hospital for diabetic ketoacidosis. He was treated and transitioned to the basal-bolus insulin regimen. LADA was suspected and later confirmed with positive glutamic acid decarboxylase 65-kilodalton isoform (GAD65) antibody. Subsequently, he was seen for follow-up at our outpatient endocrinology office. The patient stated he had been trying very hard to follow a strict diet and had met with diabetic educators. His BG levels fluctuated with activity and diet. He worked intermittently with several weeks off in between projects. His occupation involved intense physical activity as well. During a work season, his insulin dosage required frequent adjustments due to lower BG readings. In view of his variable work schedules and changing insulin needs, he was recommended a CGM device. Few days after starting on the CGM, he was seen in the emergency department because of elevated BG. His home BG readings were ranging from 80 to 408 mg/dL. He was getting frustrated with the BG readings “all over the place”. He was intentionally drinking lots of water to “help with the blood sugars”. He was concerned that his CGM device was not accurate. He felt agitated with periodically changing BG values on his CGM. He described feeling “hot” with high or low BG values. At home, he was always restless and habitually checked his CGM device. Insulin was adjusted and he was discharged with outpatient follow-up. After discharge, he was compliant with follow-up appointments. He would repeatedly call the endocrinology office with his BG readings and if needed, his insulin dose was modified accordingly. Few weeks later, the office received a call from his wife/significant other (SO) informing us that the patient had shot himself in the head. According to his wife/SO, lately, he had trouble sleeping, was very anxious, and often had panic attacks. He seemed to struggle with ever-oscillating BG readings. He was obsessed with relentlessly changing numbers and would continually look at his CGM checking for BG levels. She told us about a remote diagnosis of OCD and panic disorder in the patient. He was neither seeing a psychiatrist nor was under any treatment for it. The patient had not disclosed this information when seen at our office. We also learnt that his father had committed suicide in a similar fashion with a gun.

## Discussion

Patients with T1DM are at increased risk of mental health disorders including severe diabetes distress, depression, anxiety, eating disorders, and suicide attempts [9,11]. The benefits of using CGM in T1DM are well established [8]. However, there are only a few studies that have looked into mental health aspects associated with CGM, especially in middle-aged men let alone patients with underlying OCD and/or panic disorder. Most available literature is on children, adolescents, young adults, and parents of children with T1DM who have reported different psychological impacts of CGM use. In some, CGM use was associated with a positive, whereas in others it was associated with a negative psychosocial impact [12]. With the use of CGM, patients have reported feeling overwhelmed by the volume of data, and parents reported increased anxiety as a result of greater awareness of their child’s glucose levels [13,14]. We did not come across any reports on the impact of CGM in patients with preexisting OCD and its outcome. In this subset of patients, questions regarding calibration, precision, reliability, and variability of blood glucose levels, together with continuous ongoing alerts and alarms, can potentially result in prolonged anguish and/or worsening of OCD symptoms. Patients with T1DM patients have common fears like fluctuating BG levels, insulin dosing, nutrition, maintaining a strict lifestyle, and disease complications which can take hold and develop into persistent obsessions. Similarly, for a patient with OCD when started on CGM these fears can be augmented causing worsening of OCD symptoms.

Our patient was clearly overwhelmed by the data received from his CGM device as reported by his wife. Unfortunately, he fell victim to the negative mental health impacts of DM coupled with CGM use in the setting of a prior OCD and panic disorder. It seems logical to conclude that patients with underlying OCD when started on CGM can result in worsening of their mental health including OCD. This is a classical illustration of a Swiss cheese model first described by James Reason [15]. In the real world, safety barriers are like Swiss cheese slices; nevertheless, they can still work out fine unless the openings in several layers momentarily line up to form an accident trajectory that brings dangerous threats into contact with the victims. In the above scenario, the imperfections in the defensive barriers that may have resulted in the cumulative failure include underlying OCD, panic disorder, LADA, CGM device with recurrent reporting of BG levels, lack of psychiatry/psychosocial assessment for worsening mental health, and access to firearms [16].

Our goal in bringing this incident to light is not only to emphasize the significance of regular evaluations in patients with DM for diabetes distress and mental illness, but also to highlight the necessity for extra vigilance while delivering CGM to patients with pre-existing OCD and/or panic disorder. Thorough psychiatric history and risk assessment should be performed to determine the candidacy of CGM in OCD patients. Mental diseases are common among patients with diabetes, such as depression and suicidal ideation, yet mental health is still largely neglected in these patients [17]. There is a lack of definitive data on suicide risk associated with diabetes. A meta-analysis by Wang et al., suggested that diabetes can significantly increase the risk of suicide [18]. There is increased prevalence of depression, frequently leading to suicide in individuals with DM with a bidirectional relationship between depression and DM [19]. It accentuates the need for evaluating diabetic patients for mental illness including depression, OCD, panic disorder, and suicide risk assessment to prevent abysmal morbidity and mortality [19]. Given the increased risk of suicide in individuals with DM, the use of CGM in patients with underlying risk factors for suicide needs to be critically assessed. If started on CGM, we suggest frequent reassessment for worsening mental health and suicide risk. Periodic follow-ups are recommended after starting CGM not just to monitor BG control but also for worsening mental health including OCD. This can be done at the endocrinologist's office in conjunction with a PCP and/or psychiatrist. 

A multitude of aids are available for screening of mental health conditions. The Mini-International Neuropsychiatric Interview (MINI) is well established for structured diagnostic interviews to screen for the presence of major psychiatric disorders. Patient Health Questionnaire (PHQ)-2, PHQ-9, General Anxiety Disorder (GAD)-2, and GAD-7 are widely used and validated screening tools for depression and anxiety. They provide swift screening for a clinically significant disorder in an outpatient setting. Diabetes Distress Scale (DDS)-2 is a two-item, quick, and easily administered diabetes distress screening tool to promptly assess diabetes-specific distress. A positive DDS-2 can be followed up with DDS-17 for detailed evaluation [20]. This could help office-based providers with rapid assessment and guide them toward necessary intervention. If deemed appropriate, when initiating CGM, early involvement of patients' family/support system can be considered which can help curb additional anxiety and raise a timely alarm of worsening mental health. Overall, the objective to achieve is better patient satisfaction with CGM and to aid positively in improving morbidity and mortality along with averting any untoward outcomes.

## Conclusions

Suicide should not be an outcome for any disease. This case highlights the importance of considering mental health as a prognostic factor in the management of DM. We suggest obtaining detailed psychiatric history along with evaluation for underlying OCD, panic disorder, and suicide risk assessment in all patients planned for CGM. Cautious planning is recommended prior to starting CGM in OCD patients to ward off catastrophic consequences. Limited data is available on the use of CGM in patients with psychiatric comorbidities, especially the adult population. Further research and detailed studies are required to establish guidelines for the use of CGM in such patients.

## Appendices

**Table 1:** Comparison of LADA and T1DM GAD65*: Glutamic acid decarboxylase 65-kilodalton isoform antibody; ICA**: islet cell autoantibodies; IA2***: tyrosine phosphatase–related islet antigen 2; IAA****: insulin autoantibodies; LADA: latent autoimmune diabetes in adults; T1DM: type 1 diabetes mellitus Source: Data from [7]

	LADA	T1DM
Age (years)	≥30	<35
C-peptide	Low	Low
Circulating Insulin	Gradual deficiency	Rapid deficiency
Timeline (diagnosis to insulin requirement)	Variable (usually within six months)	At diagnosis
GAD65*	can be positive	usually positive
ICA**	can be positive	usually positive
IA2***	can be positive	usually positive
IAA****	can be positive	usually positive
